# Exploring the Impact of Race/Ethnicity Match on Relationship Quality: Incorporating Parent and Home Visitor Perspectives

**DOI:** 10.1007/s10995-025-04206-3

**Published:** 2026-01-06

**Authors:** Sandra Tang, Robin Jacob, Megan Foster Friedman

**Affiliations:** Institute for Social Research, University of Michigan, 426 Thompson St, Ann Arbor, MI 48104 USA

**Keywords:** Race/Ethnicity, Home visiting, Relationship quality

## Abstract

**Objective:**

The primary goal of this study is to investigate whether a race/ethnicity match between parents and home visitors (HV) is associated with their relationship quality and whether quality ratings differ depending on parent or HV report.

**Methods:**

Participants included 1,461 parent/HV dyads who participated in the Maternal Infant Health Program, Michigan’s largest evidence-based home visiting program for Medicaid-eligible pregnant people and infants. At the time of discharge, parents and HVs were asked to complete a brief survey and reported on their background, program experiences, and relationship quality. We ran a series of multivariable logistic regression analyses and regressed HV- and parent-reported relationship quality ratings on race/ethnicity (mis)match controlling for covariates that likely contribute to the HV-parent relationship quality.

**Main Findings:**

Both parents and HVs rated the relationship quality positively, but their ratings were only moderately correlated, which suggests that parents and HVs use different criteria to determine relationship quality. When there is a race/ethnicity match, HVs rate the relationship quality higher, but match is not associated with parents’ relationship quality ratings. In general, Black parents reported lower relationship quality ratings compared to White parents, which suggests that Black parents’ dissatisfaction is stemming from sources other than a race/ethnicity mismatch.

## Significance

An important factor that drives families’ participation and retention in maternal and infant home visiting programs is the HV-parent relationship quality. To foster positive relationships with families’ of minoritized racial/ethnic backgrounds, researchers and practitioners underscore the importance of service providers demonstrating cultural competence. Currently, there is limited work empirically exploring this area. The present study fills this gap by investigating whether a race/ethnicity match between parents and home visitors is associated with their relationship quality. Results indicate that parents and home visitors use different rating criteria and highlight the importance of considering both perspectives to understand relationship quality.

Maternal and infant home visiting programs—in which a professional home visitor (HV), such as a nurse or social worker, engages with the family at their residence and connects them to services and resources that help address economic, social, and health-related challenges—have received considerable interest and financial investment in recent years. Although there are a variety of home visiting program models, all emphasize a two-generation, family-centered approach. Research indicates that home visiting programs can improve maternal and infant health, support positive parenting, and promote children’s school readiness (ACF & HRSA, 2016). The overall program effects, however, are modest and inconsistent across studies, leading researchers to advocate for investigating individual program components separately and identifying ways to increase their quality (Filene et al., 2013; Korfmacher et al., 2019). Further, these programs are often underutilized, with low levels of program take-up and persistence (Duggan et al., 2018). More research is needed to identify ways to improve engagement and ongoing participation in these programs.

One key factor that drives families’ participation and retention in home visiting programs is the relationship quality between the HV and parent. Relationship quality indicates the level of satisfaction with the interaction, support provided, and connection between the family and program staff (Korfmacher et al., 2008). Prior research finds that higher quality relationships are linked to greater participation and longer retention in home visiting programs (Bower et al., 2020; Brookes et al., 2006; Kleinman et al., 2023; Manz & Ventresco, 2019). For parents who may be mistrustful of service providers, such as those experiencing poverty and from racial/ethnic-minority backgrounds, a positive relationship is particularly important for facilitating engagement (Korfmacher et al., 2007; Manz & Ventresco, 2019).

A variety of factors contribute to developing positive relationships: mutual trust, respect and knowledge of families’ strengths and goals, and understanding of the context in which families exist and make decisions (AAP, 2003; Kleinman et al., 2023; Wasik, 1993). To foster positive relationships, especially with families of minoritized racial/ethnic backgrounds, family-centered early intervention programs underscore the importance of service providers demonstrating cultural competence and accommodating families’ linguistic preferences, which in turn, empowers families to make decisions and fosters a collaborative partnership between the service provider and family (AAP, 2003; Blue-Banning et al., 2004). Indeed, parents report greater satisfaction when HVs exhibit cultural sensitivity and provide culturally appropriate support (Damashek et al., 2012; Kleinman et al., 2023).

Together this suggests that a match between the race/ethnicity of a HV and a parent may be an important factor that leads to more positive relationships and potentially to increased levels of participation. HVs paired with parents who share a similar racial/ethnic background are more likely to have a common cultural perspective (e.g., similar values regarding parenting, relationships, goals, etc.) which can foster their ability to engage in culturally appropriate interactions and conversations. In turn, parents may feel more connected to and trusting of the HV. When there is a race/ethnicity match, HVs may also be less likely to hold stereotypes or biases that influence their assessment of family needs and referral decisions (Gilliam et al., 2016; McGrady & Reynolds, 2013). Finally, HVs who share a racial/ethnic background with parents may be more adept in the role of a cultural broker because they may be more effective at bridging cultural gaps and facilitating families’ access to resources that are sensitive to their cultural needs and aligned with their priorities.

Despite these hypothesized benefits, to date, there has been limited work to empirically explore whether having a racial/ethnic match between HVs and families leads to better relationships or increased participation. We were only able to identify a few studies that address this topic. One study exploring home visiting programs in Tribal communities found program recruitment to be more effective when the demographic characteristics of HVs and parents were aligned, and when HVs incorporated families’ cultural and social norms in their approach for addressing family needs (Barlow et al., 2018). Another study found that *programs* that had greater success matching HVs with families on race/ethnicity had families who participated in more home visits and stayed in the program longer, but this study did not focus on the race/ethnicity match of individual family/HV dyads (Daro et al., 2003). A separate study, using the same sample, found that race/ethnicity match did not influence retention for White or Latino families, but did matter for Black families (McCurdy et al., 2003). Given these limited and inconsistent findings, more research on the benefits of pairing HVs and parents according to their racial/ethnic backgrounds is needed (Azzi-Lessing, 2013; Bower et al., 2020; Kleinman et al., 2023).

## Methods

The primary goal of this study is to investigate whether a race/ethnicity match between parents and HVs is associated with the parent and HV relationship quality ratings and whether this differs for parents and HVs. We also explore whether a race/ethnicity match predicts the likelihood that parents complete the home visiting program, as evidenced by whether they completed a survey during a discharge appointment.

### Participants

The participants for the present study included 1,461 parent and HV dyads who participated in the Maternal Infant Health Program (MIHP), Michigan’s largest evidence-based home visiting program for Medicaid-eligible pregnant people and infants. These dyads participated in the *Healthy Moms, Healthy Babies MIHP Pilot Project*, a randomized control trial evaluation of an intervention with over 50 MIHP provider agencies across the state that was conducted by the Michigan Department of Health and Human Services and the study authors at the University of Michigan Youth Policy Lab. At the time of discharge from MIHP, parents and HVs were asked to complete a brief survey. Parents and HVs reported on their background and experiences and interactions with one another, including their relationship quality. The study sample was restricted to dyads where both the parent and HV submitted an exit survey upon discharge from the program. This study (HUM00195929) was reviewed by the University of Michigan Institutional Review Board and conducted in accordance with prevailing ethical principles. All study participants gave their informed consent prior to their inclusion in the study.

### Measures

#### Excellent Relationship Rating

Parents and HVs reported on the quality of their relationship with one another (*“How would you rate your relationship with this Home visitor?*”; “*How would you rate your relationship with this beneficiary?”*) using the response options of *Excellent*, *Very good*, *Good*, *Okay*, and *Poor*. Since ratings across both groups were overwhelmingly positive, a 0/1 dichotomous variable was created where 1 indicates an *Excellent* rating and 0 indicates a rating less than *Excellent*. In the full sample, 70% of parents and 49% of HVs rated their relationship as *Excellent*.

#### Race/Ethnicity Match

HVs reported on parents’ and their own race/ethnicity background. Although parents also reported on their own race/ethnicity background, we used HV reports because there were fewer missing data and reports were highly correlated (*r *> 0.75). A 0/1 dichotomous variable was created where 1 indicates a match between the race/ethnicity of the parent and HV. In the full sample, 53% of the dyads had a race/ethnicity match.

#### Covariates

A variety of other factors (e.g., parent characteristics such as race/ethnicity, first pregnancy, partnership status, and mental health; or the HV’s ability to link families to services) may contribute to the quality of the HV-parent relationship. For example, a first-time or anxious parent may welcome the extra support that an HV can provide whereas a depressed parent may want to limit interactions with an HV, which can impede building a trusting relationship (Bower et al., 2020). Relationship quality may also depend on the perceived utility of the relationship, such as the HV’s ability to connect parents with resources. Additionally, agency-level factors (e.g., organizational culture, resources and support, policies, and procedures, etc.) shape the context in which the HV-parent relationship operates, which may affect their relationship quality (Gomby, 2007). To account for these factors, we include the following covariates in our statistical models: 

#### Parent’s Race/Ethnicity

HVs reported on parents’ race/ethnicity background. Dichotomous variables (0/1) were created for the following groups: Black, Latino, White, and Other. The Other group consisted of parents who were American Indian/Alaska Native, Arab/Chaldean, Asian, Multiracial, or Native Hawaiian or Other Pacific Islander. In the full sample, 31% of the parents were Black, 6% Latino, 13% Other, and 49% White.

#### First Pregnancy

Based on parent report, a 0/1 dichotomous variable was created to indicate if this was the parent’s first pregnancy. In the full sample, 37% reported this as their first pregnancy.

#### Parent has a Partner

Parents reported on their partner status by answering the question: “*Are you in a relationship right now?*”. A 0/1 dichotomous variable was created where 1 indicates the parent had a partner. 73% of the full sample were partnered.

#### Symptoms of Anxiety

Parents answered questions from the Generalized Anxiety Disorder-2 (GAD-2; Kroenke et al., 2007), which is a pre-screener for a generalized anxiety disorder. They responded to two items: “*In the past 2 weeks, how often have you been bothered by the following problems: feeling nervous, anxious or on edge; not being able to stop or control worrying?*” on a 4-point scale (0-*Not at all*, 1-*Several days*, 2-*More than half the days*, 3-*Nearly every day*). A 0/1 dichotomous variable was created where 1 indicates parents who scored three or more points on the two items, which is the recommended cutoff for further diagnostic evaluation. In the full sample, 16% of parents exhibited symptoms of anxiety.

#### Symptoms of Depression

Parents answered questions from the Patient Health Questionnaire-2 (PHQ-2; Kroenke et al., 2003), which is a pre-screener for a depressive disorder. They responded to two items: “*In the past 2 weeks, how often have you been bothered by the following problems: little interest or pleasure in doing things; feeling down, depressed, or hopeless?*” on a 4-point scale (0-*Not at all*, 1-*Several days*, 2-*More than half the days*, 3-*Nearly every day*). A 0/1 dichotomous variable was created where 1 indicates a parent who scored three or more points, which is the recommended cutoff for further diagnostic evaluation. In the full sample, 10% of parents exhibited symptoms of a depressive disorder.

#### Number of Referrals

Parents and HVs reported on the number of distinct categories of referrals for additional support (e.g., food, housing, transportation, mental health, etc.) that were made while participating in MIHP. The number of referrals ranged from 0 to 8, with HVs reporting a mean of 0.75 (SD = 1.26) and parents reporting a mean of 1.76 (SD = 1.80) referrals in the full sample.

#### MIHP Agency Type

Three indicator variables were created to represent the types of MIHP provider agencies in the sample: federally qualified health center (FQHC) and health system (17%); independent agency (26%); and local health department (57%). Independent agencies are typically run by individuals or small non-profits and are not affiliated with a hospital system or health department.

#### Treatment

A dichotomous 0/1 variable was created to indicate if the parent was in the study’s treatment group. In the full sample, 72% of the parents were in the treatment group.

### Analytic approach

To answer our research questions, we ran a series of multivariable logistic regression analyses. We regressed HVs’ and parents’ *Excellent* relationship quality ratings on the covariates listed above and race/ethnicity (mis)match as a predictor variable.

Across all variables, there was a relatively low to moderate amount of missing data, and data with missing values were excluded from our analyses using listwise deletion. Parents who were Black, Latino, had symptoms of anxiety, and respondents from independent agencies were more likely than their counterparts to be excluded from the analytic sample. In contrast, HVs who rated the relationship quality as *Excellent*, dyads with a race/ethnicity match, White parents, and respondents who reported more referrals and who received services from independent agencies were more likely than their counterparts to be in the sample.

## Results

Table 1 displays descriptive statistics for all study variables in the full sample and race/ethnicity (mis)match subgroups. Most parents (70%) rated their relationship quality as *Excellent* whereas less than half of HVs (49%) did so. Correlations (not shown) indicate that HV and parent quality ratings are modestly correlated (*r* = 0.41). Bivariate regression models testing group differences indicate that HVs are more likely to rate the relationship quality as *Excellent* when there is a race/ethnicity match. Parents in the match group are more likely to be White, have a partner, exhibit symptoms of anxiety and depression, and receive services from an independent agency. Parents in the mismatch group are more likely to be non-White, report a higher number of parent-reported referrals, receive services from an FQHC or health system, and to be in the treatment group.

**Table 1:** Descriptive statistics for study variables

	Total Sample (N = 1461)			Race/Ethnicity Match (n = 636)			Race/Ethnicity Mismatch (n = 528)		
	M / %	*SD*	% Missing	M / %	*SD*	% Missing	M / %	*SD*	% Missing
Excellent relationship, Home visitor rating*	48.94%		9.65%	50.71%		0.47%	44.51%		0.00%
Excellent relationship, Family rating	70.40%		6.57%	71.29%		0.31%	70.69%		1.14%
Race/Ethnicity match*	54.64%		20.33%	100.00%		0.00%	0.00%		0.00%
Parent: Black*	31.39%		19.10%	13.52%		0.00%	52.65%		0.00%
Parent: Latino*	5.92%		19.10%	1.10%		0.00%	11.93%		0.00%
Parent: Other*	13.28%		19.10%	2.52%		0.00%	26.52%		0.00%
Parent: White*	49.41%		19.10%	82.86%		0.00%	8.90%		0.00%
First Pregnancy	36.77%		5.61%	37.76%		0.47%	35.81%		0.57%
Parent has a partner*	72.91%		6.78%	77.64%		1.57%	66.60%		0.76%
Family has symptoms of anxiety*	15.60%		13.14%	18.44%		7.08%	11.18%		8.52%
Family has symptoms of depression*	9.79%		12.59%	11.24%		6.29%	7.01%		8.14%
Number of referrals reported by HV	0.75	1.26	0.00%	0.88	1.38	0.00%	0.83	1.30	0.00%
Number of referrals reported by Family*	1.76	1.80	0.00%	1.73	1.74	0.00%	2.02	1.88	0.00%
FQHC and Health system*	17.32%		5.54%	13.36%		0.00%	25.19%		0.00%
Independent agency*	25.87%		5.54%	28.93%		0.00%	21.78%		0.00%
Local health department agency	56.81%		5.54%	57.70%		0.00%	53.03%		0.00%
Treatment*	71.74%		5.54%	62.74%		0.00%	85.23%		0.00%

Asterisks indicate statistically significant differences (p < 0.05) between race/ethnicity match and mismatch groups

### Multivariable Logistic Regression Models

Table 2 presents the results from multivariable logistic regression models that regressed race/ethnicity match, parents’ race/ethnicity, first pregnancy, partner status, mental health, number of referrals reported by the HV and parent, agency type on both HVs’ (first set of columns) and parents’ (second set of columns) *Excellent* relationship quality ratings. For HVs, when there is a race/ethnicity match, HVs have 80% higher odds of rating the relationship quality as *Excellent*. Similarly, HVs have 120% higher odds of rating the relationship as *Excellent* when working with parents who have a race/ethnicity background of Other vs. White. For each additional referral reported by the HV, the odds of HVs rating the relationship quality as *Excellent* increases by 12%. HVs in FQHCs, health systems, and independent agencies have lower odds of rating their relationship quality as *Excellent* compared to HVs in local health departments.

**Table 2:** Multivariable logistic regression models predicting excellent relationship quality rating

	Home Visitors’ Rating (n = 1051)						Parents’ Rating (n = 1052)					
	Adjusted OR	SE	*z*	*p*	[95%	CI]	Adjusted OR	SE	*z*	*p*	[95%	CI]
Race/Ethnicity match	1.80	0.37	2.81	.01	1.19	2.70	0.71	0.15	−1.6	.11	0.47	1.08
Parent: Black	1.44	0.31	1.69	.09	0.94	2.19	0.62	0.14	−2.12	.03	0.40	0.96
Parent: Latino	1.21	0.40	0.60	.55	0.64	2.30	0.51	0.18	−1.93	.05	0.26	1.01
Parent: Other	2.20	0.58	2.98	.00	1.31	3.68	0.97	0.28	−0.12	.91	0.55	1.70
First pregnancy	0.98	0.13	−0.14	.89	0.76	1.27	0.95	0.14	−0.37	.71	0.71	1.26
Parent has a partner	1.22	0.18	1.31	.19	0.91	1.63	1.83	0.29	3.79	.00	1.34	2.49
Family has symptoms of anxiety	1.03	0.20	0.16	.88	0.70	1.52	1.03	0.23	0.15	.88	0.67	1.60
Family has symptoms of depression	1.29	0.32	1.04	.30	0.80	2.09	1.14	0.32	0.49	.63	0.66	1.97
Number of referrals reported by HV	1.12	0.06	2.20	.03	1.01	1.23	1.14	0.07	2.17	.03	1.01	1.27
Number of referrals reported by family	1.07	0.04	1.71	.09	0.99	1.15	1.09	0.05	1.91	.06	1.00	1.18
FQHC and health system	0.61	0.11	−2.72	.01	0.42	0.87	0.80	0.16	−1.08	.28	0.54	1.20
Independent agency	0.52	0.08	−4.06	.00	0.37	0.71	0.36	0.06	−5.88	.00	0.26	0.51
Treatment	0.92	0.14	−0.53	.60	0.68	1.25	0.83	0.15	−1.04	.30	0.59	1.17
Constant	0.49	0.14	−2.43	.02	0.28	0.87	2.85	0.88	3.37	.00	1.55	5.23
Likelihood ratio of Chi-squared test	43.68						76.53					
P-value	.00						.00					

OR = Odds Ratio

In the model predicting parents’ relationship ratings (second set of columns), race/ethnicity match did not predict parents’ *Excellent* relationship ratings, but Black parents have 38% lower odds of rating the relationship quality as *Excellent* compared to White parents. In contrast, partnered parents have 83% higher odds of rating the relationship as *Excellent* than non-partnered parents. For each additional referral reported by the HV, the odds of parents rating the relationship as *Excellent* increases by 14%. Finally, families served by independent agencies have lower odds of rating their relationship quality as *Excellent* compared to parents served by local health departments.

Given the significant finding for Black parents, we conducted exploratory analyses and re-ran the models restricted to Black parents (see Table 3). Black HV’s had 140% higher odds of rating the relationship quality as *Excellent* when they worked with Black families. In contrast, race/ethnicity match had no significant bearing on Black families’ relationship quality ratings. In models restricted to White parents (not shown), a race/ethnicity match had no significant association with either group’s relationship quality ratings.

**Table 3:** Multivariable logistic regression models predicting excellent relationship quality rating for Black families

	Home Visitors’ Rating (n = 319)						Parents’ Rating (n = 319)					
	Adjusted OR	SE	*z*	*p*	[95%	CI]	Adjusted OR	SE	*z*	*p*	[95%	CI]
Race/Ethnicity match	2.39	0.80	2.60	.01	1.24	4.60	0.61	0.20	−1.55	.12	0.32	1.14
First pregnancy	0.98	0.25	−0.09	.93	0.60	1.61	0.88	0.23	−0.47	.64	0.53	1.48
Parent has a partner	0.83	0.20	−0.78	.44	0.51	1.33	1.78	0.45	2.28	.02	1.08	2.93
Family has symptoms of anxiety	0.66	0.28	−0.97	.33	0.29	1.52	0.51	0.22	−1.58	.12	0.22	1.18
Family has symptoms of depression	1.17	0.57	0.33	.74	0.45	3.02	1.80	0.91	1.17	.24	0.67	4.86
Number of referrals reported by HV	1.03	0.09	0.31	.76	0.87	1.22	1.10	0.11	0.95	.34	0.91	1.32
Number of referrals reported by Family	1.15	0.08	2.02	.04	1.00	1.32	1.26	0.10	2.94	.00	1.08	1.48
FQHC and health system	0.54	0.16	−2.05	.04	0.30	0.97	0.60	0.20	−1.55	.12	0.32	1.15
Independent agency	0.28	0.09	−3.81	.00	0.15	0.54	0.29	0.10	−3.72	.00	0.15	0.56
Treatment	1.97	0.81	1.65	.10	0.88	4.40	0.63	0.26	−1.11	.27	0.28	1.42
Constant	0.52	0.23	−1.48	.14	0.22	1.24	2.30	1.07	1.80	.07	0.93	5.72
Likelihood ratio of Chi-squared test	24.73						42.21					
P-value	.01						.00					

OR = Odds Ratio

## Discussion

The present study examined whether having a race/ethnicity match between a parent and HV was associated with their relationship quality. Overall, both parents and HVs rated the relationship quality positively, but their *Excellent* ratings were only moderately correlated, which suggests that parents and HVs use different criteria to determine high quality relationships and highlights the importance of examining both perspectives.

### Race/Ethnicity Match

Race/ethnicity match was positively and statistically significantly associated with relationship quality ratings for HVs but not for parents. These associations remained even when the sample was restricted to Black parents. The results indicate that Black HVs are more likely to rate their relationship quality high if they are serving Black parents. It may be that Black HVs feel more uncomfortable serving families of different races. They also may have received training that makes them more aware of power dynamics, or cultural differences that impact the quality of their relationships. In the health care literature, studies document that physicians’ beliefs about and expectations of the patient are tied to patients’ race/ethnicity (Porter & Beuf, 2010; van Ryn & Burke, 2000). Thus, when there is a race/ethnicity background match, HVs may feel like they are able to align their beliefs and expectations of the parent with their communication style and approach to addressing parents’ needs.

Conversely, there is no statistically significant difference in the way that parents rate the relationship quality with their HV regardless of whether there is a race/ethnicity match. In sensitivity analyses, we investigated whether this is because parents who were most dissatisfied with their HVs dropped out of the program and did not complete an end of program survey. Results (not shown here) indicate that race/ethnicity match does not predict whether a parent completed an end of program survey, thus discrediting the idea that the parents with a race/ethnicity mismatch who were dissatisfied with their relationship with their HVs are not represented in the data. This finding—that a race/ethnicity match does not matter for parents’ relationship quality rating—is counter to extant research using data from the 1994 Commonwealth Fund Minority Health Survey, which found that when there was patient-provider race concordance, patients, especially African American patients, were more likely to use needed health services (LaVeist, Nuru-Heter, & Jones, 2003), and experienced more satisfaction with their physician (LaVeist & Carroll, 2002; Saha et al., 1999). Similarly, Cooper et al. (2003) found that patients rated their health care provider more highly when their racial background matched with their provider’s background.

In the present study, however, it appears that parents are more focused on the support they receive and less on the racial/ethnic background of their HV. As suggested in other research, it is likely that parents who experience poverty and elect to participate in a home visiting program are overwhelmed and stressed, and just having consistent access to a professional who can provide advice and support is highly valued (Daro et al., 2003; Pharis & Levin, 1991). The difference between our finding and prior literature may also reflect cohort differences. The LaVeist et al. (2003) and Saha et al. (1999) studies used data from over twenty years ago and parents at that time may have had different care provider preferences than parents today. It may be that parents today place greater value in having alignment in other areas, such as the HV’s personality, personal history, or cultural competence. For example, parents have reported feeling a stronger bond with HVs who are conscientious, follow through on their promises, have children, and/or experienced similar hardships (Brookes et al., 2006). Additionally, in the health care literature, patients report greater satisfaction with their provider when they feel like they are part of a partnership and are involved with the medical decision-making process (Cooper & Roter, 2003), which may occur regardless of whether there is a race/ethnicity match.

Although race/ethnicity match is not significantly associated with parents’ relationship quality ratings, Black parents in this study rate their relationship quality lower than White parents after accounting for race/ethnicity match. This finding aligns with recent survey results where Black adults were more likely to report negative health care experiences and feel that they are treated unfairly or with disrespect due to their race/ethnicity background compared to their White counterparts (Artiga, Hill & Presiado, 2024). This suggests that any dissatisfaction stems from sources other than a race/ethnicity match. It may be that Black parents are being served by agencies with the least resources, and/or with less skilled HVs. It could also be that these parents have more complicated home situations that are difficult for the HV to address effectively, leading to dissatisfaction.

### Other Predictors of Relationship Quality

The number of referrals reported by the HV is positive and statistically significantly associated with the relationship quality ratings of both HVs and parents. This suggests that HVs feel good about being able to connect families with services, and that parents appreciate the service referrals provided by the program. Corroborating this idea is research by Roggman and colleagues (2001) who found that HVs rated the quality of their home visits and relationship with families more positively when they felt like families benefitted from the program. For Black parents, the number of referrals *reported by the parent* was positively and statistically significantly associated with the relationship quality ratings of both HVs and parents. The point estimate for parent-reported referrals increased quite a bit from the full model to the restricted model, which suggests that Black parents place high value on the referrals, potentially because they are in greater need.

Partnered parents are statistically significantly more likely than non-partnered parents to rate their relationship quality with the HV highly. It may be that HVs help parents have more positive relationships with their partner by encouraging both to take part in parenting responsibilities. Other research also suggests that parents who have supportive, positive family relationships may more easily develop positive relationships with the HV (Brookes et al., 2006).

Finally, agency type plays a consistent statistically significant role in relationship quality. The relationship quality among independent agencies was rated lower by both HVs and parents relative to agencies run by local health departments. MIHP agencies that are affiliated with larger health care organizations (e.g., local health departments, health systems, or FQHCs) are often able to draw upon other resources, services, funding opportunities, and/or staffing infrastructure offered by their parent organization. For example, a referral from MIHP to WIC is much easier to complete when the two offices are located on the same floor of the same health department office. In contrast, many independent agencies exist for the sole purpose of providing MIHP home visiting services, are not affiliated with broader health care organizations, and operate similarly to a small business.

Independent agencies often have the fewest resources available to them to serve families and this may be reflected in the relationship quality ratings. Fewer resources can mean less time spent with families, less time available for follow-up and care coordination, potentially lower salaries for staff, which could mean HVs with less experience, less training, or fewer skills, which could be related to satisfaction among HVs. Similarly, FQHCs and health systems also had lower relationship quality ratings among HVs compared to local health departments. FQHCs and health systems may be more bureaucratic than local health departments, with goals that are not as well aligned with home visiting services, which might make it harder for HVs to do their jobs as well as they would like.

### Limitations and Conclusions

This study has several limitations. First, not every parent and HV who participated in the home visiting program completed a survey. In sensitivity analyses (results not presented), we did not find any statistically significant differences in the proportion of race/ethnicity match in our sample compared to those who were excluded from the analysis, and parents were just as likely to have completed a survey regardless of whether there was a race/ethnicity match or mismatch. However, non-White parents were less likely to complete a survey, which may have led to potential bias in study findings due to survey non-response. For example, if the families who did not respond, or had missing data, were less satisfied than families with complete data, we might be underestimating the level of dissatisfaction among families.

Two, although this study incorporated both parent and HV perspectives, only quantitative measures of relationship quality were studied. Extant literature suggests that parent and HVs tend to rate their relationship quality more positively when responding to quantitative survey items compared to qualitative, open-ended interviews (Korfmacher & Marchi, 2002). Future studies that investigate how a race/ethnicity match affects relationship quality should incorporate qualitative responses for a more comprehensive understanding of when and for whom a race/ethnicity match matters.

Despite these limitations, this study has several strengths. It incorporates the perspectives of both the parent and HV, which allows for a more comprehensive understanding of how a race/ethnicity match is associated with relationship quality. Investigating multiple perspectives is particularly important for understanding a construct like relationship quality, which is multifaceted and complex, and hinges on factors related to various parties and contexts (Manz & Ventresco, 2019). Importantly, this study indicates that parents’ ratings of relationship quality are less sensitive to race/ethnicity match than HVs’ and suggests that more research is needed on the factors that parents use to determine a high-quality HV-parent relationship, which ultimately contributes to their engagement in home visiting programs (Brookes et al., 2006). Results also indicate that HVs’ relationship quality ratings are tied to their sense of professional competence and ability to support families’ needs. Given that agency characteristics shape the context in which HV-parent relationships operate, it is important to investigate which structural elements support or hinder the development of a high-quality relationship between parents and HVs across racial/ethnic backgrounds.

As noted in prior research, relationship quality is a key factor that contributes to families’ participation and retention in home visiting programs. The present study adds to this literature by highlighting that families and HVs may be using different criteria to assess relationship quality, which in turn, may affect program participation and retention. Thus, HV trainings should raise awareness that families’ assessment of their relationship quality may differ from HVs’ assessment and provide different strategies to help HVs with connecting and building trust with families to promote a positive relationship.

## Data Availability

The authors are unable to share the data publicly due to terms of the IRB and the data sharing agreement with the state of Michigan.
